# Cinnamaldehyde and cinnamaldehyde derivatives reduce virulence in *Vibrio *spp. by decreasing the DNA-binding activity of the quorum sensing response regulator LuxR

**DOI:** 10.1186/1471-2180-8-149

**Published:** 2008-09-16

**Authors:** Gilles Brackman, Tom Defoirdt, Carol Miyamoto, Peter Bossier, Serge Van Calenbergh, Hans Nelis, Tom Coenye

**Affiliations:** 1 Laboratory of Pharmaceutical Microbiology, Ghent University, Harelbekestraat 72, B-9000 Ghent, Belgium; 2 Laboratory of Microbial Ecology and Technology, Ghent University, Coupure Links 653, 9000 Ghent, Belgium; 3 Laboratory of Aquaculture and Artemia Reference Center, Ghent University, Rozier 44, 9000 Ghent, Belgium; 4 Department of Biochemistry, McGill University, McIntyre Medical Sciences Building, Room 818, 3655 Promenade Sir William Osler, Montreal, H3G 1Y6, Canada; 5 Laboratory of Medicinal Chemistry, Ghent University, Harelbekestraat 72, B-9000 Ghent, Belgium

## Abstract

**Background:**

To date, only few compounds targeting the AI-2 based quorum sensing (QS) system are known. In the present study, we screened cinnamaldehyde and substituted cinnamaldehydes for their ability to interfere with AI-2 based QS. The mechanism of QS inhibition was elucidated by measuring the effect on bioluminescence in several *Vibrio harveyi *mutants. We also studied *in vitro *the ability of these compounds to interfere with biofilm formation, stress response and virulence of *Vibrio *spp. The compounds were also evaluated in an *in vivo *assay measuring the reduction of *Vibrio harveyi *virulence towards *Artemia *shrimp.

**Results:**

Our results indicate that cinnamaldehyde and several substituted derivatives interfere with AI-2 based QS without inhibiting bacterial growth. The active compounds neither interfered with the bioluminescence system as such, nor with the production of AI-2. Study of the effect in various mutants suggested that the target protein is LuxR. Mobility shift assays revealed a decreased DNA-binding ability of LuxR. The compounds were further shown to (i) inhibit biofilm formation in several *Vibrio *spp., (ii) result in a reduced ability to survive starvation and antibiotic treatment, (iii) reduce pigment and protease production in *Vibrio anguillarum *and (iv) protect gnotobiotic *Artemia *shrimp against virulent *Vibrio harveyi *BB120.

**Conclusion:**

Cinnamaldehyde and cinnamaldehyde derivatives interfere with AI-2 based QS in various *Vibrio *spp. by decreasing the DNA-binding ability of LuxR. The use of these compounds resulted in several marked phenotypic changes, including reduced virulence and increased susceptibility to stress. Since inhibitors of AI-2 based quorum sensing are rare, and considering the role of AI-2 in several processes these compounds may be useful leads towards antipathogenic drugs.

## Background

Vibriosis, caused by *Vibrio *spp., is a major disease of marine fish and shellfish and is an important cause of economic loss in aquaculture [1,2]. Until recently prophylactic antibiotics were extensively used in aquaculture [3,4]. However, overuse of antibiotics resulted in increased rates of resistance so that novel approaches are required to manage vibriosis [5]. A possible novel target is the bacterial communication system. Bacteria monitor their population densities through the production and sensing of small signal molecules called autoinducers, a process entitled as quorum sensing (QS). To date three types of QS systems have been identified in *Vibrio *spp. [6]. Acylated homoserine lactones (AHL) are used as signal molecules in the LuxM/N QS system [7], while in the CqsA/S system, (S)-3-hydroxytridecan-4-one ("Cholera autoinducer 1", CAI-1) is used [8]. A third QS system appears to be shared by many Gram-positive and Gram-negative bacteria and is based on a mixture of interconvertible molecules collectively referred to as autoinducer-2 (AI-2) [9]. A key enzyme in the production of AI-2 is LuxS. LuxS catalyzes the cleavage of S-ribosylhomocysteine to homocysteine and 4,5-dihydroxy-2,3-pentanedione (DPD) [10]. DPD will subsequently undergo spontaneous derivatizations, forming a mixture of molecules, including (2R,4S)-2-methyl-2,3,3,4-tetrahydroxytetrahydrofuran (R-THMF) and (2S,4S)-2-methyl-2,3,3,4-tetrahydroxytetrahydrofuran-borate (S-THMF-borate) [11]. Although not all QS systems are present in all *Vibrio *spp., most of them contain the AI-2 based QS system [12]. In *Vibrio *spp. AI-2 binds to LuxP, a periplasmic AI-2 receptor that is associated with the LuxQ sensor kinase-phosphatase. At low population density only basal amounts of diffusible signal molecules are produced, and in this situation LuxQ will act as a kinase resulting in a phosphorylation of the response regulator LuxO through a cascade involving LuxU. Phosphorylation activates LuxO resulting in the production of small regulatory RNAs [13]. These small RNAs, together with the chaperone protein Hfq, will destabilize mRNA encoding the response regulator LuxR. However, when population density is sufficiently high, AI-2 will bind to LuxP and as a result LuxQ will act as a phosphatase, leading to a dephosphorylation of LuxO [14]. Since dephosphorylated LuxO is inactive, no small regulatory RNAs will be formed and the LuxR mRNA remains stable, resulting in the production of LuxR and ultimately an altered gene expression pattern. AI-2 based QS was found to play an important role in regulating the production of several virulence factors, biofilm formation and stress responses in several *Vibrio *spp. [15-17] and it was found to be associated with virulence as shown in several *in vivo *assays [18,19]. In contrast, in *Vibrio cholerae*, CAI-1 was found to be the principle signal molecule in virulence regulation [8]. Because of this involvement in virulence, inhibitors of AI-2 based QS have been proposed as novel antipathogenic agents. While there is a growing interest in and evidence for the use of these antipathogenic substances to interfere with interspecies QS in the control of virulence and biofilm formation, only a few inhibitors of AI-2 based QS are known, including halogenated furanones and cinnamaldehyde [20-23]. Halogenated furanones have previously been shown to disrupt AHL and AI-2 based quorum sensing in *Vibrio *spp. by decreasing the DNA-binding activity of the response regulator LuxR [24-26]. Halogenated furanones can attenuate the virulence of several *Vibrio *spp. in gnotobiotic brine shrimp *Artemia franciscana *and their use results in a reversal of the negative effects of *Vibrio harveyi *BB120 towards rotifers [27,28]. Unfortunately, the toxicity of halogenated furanones towards both brine shrimp and rotifers limits their use. In contrast, cinnamaldehyde is a non-toxic synthetic flavouring substance that is widely used in food, beverages, chewing gum, and the perfume and food chemistry, and is generally recognised as safe [29]. Cinnamaldehyde concentrations in food range from 4 ppm to more than 300 ppm [30]. Although cinnamaldehyde is known to be a QS-inhibitor [21], its exact mechanism of action remains to be elucidated. The goal of the present study was to determine the mechanism of action of cinnamaldehyde and to evaluate its effect on virulence of *Vibrio *spp. *in vitro *and *in vivo*.

## Results and discussion

### Effect of cinnamaldehyde and cinnamaldehyde derivatives on microbial growth

**Figure 1:**
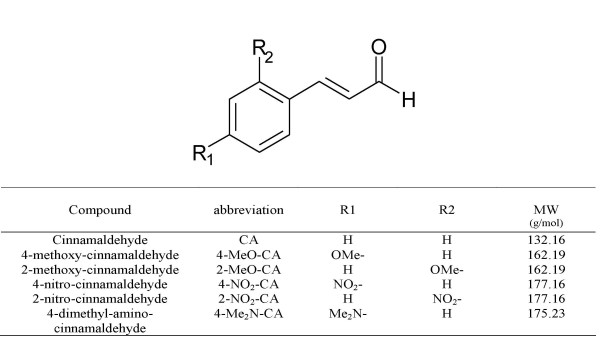
Cinnamaldehyde and cinnamaldehyde derivatives used in this study.

When used in concentrations up to 150 μM, cinnamaldehyde and most cinnamaldehyde derivatives (Fig. 1) had no inhibitory effect on the growth of strains in the present study (data not shown). The same was true for 4-NO_2_-cinnamaldehyde, but only in concentrations up to 50 μM. In all further experiments, 100 μM was used (except for 4-NO_2_-cinnamaldehyde, 25 μM), unless otherwise mentioned.

### Effect of cinnamaldehyde and 2-NO_2_-cinnamaldehyde on bioluminescence

To rule out direct interference with the bioluminescence system of *Vibrio harveyi*, a constitutively bioluminescent strain was constructed. A plasmid containing *luxCDABE *genes under *lacZ *promotion was conjugated into *Escherichia coli *DH5α (a strain defective in AI-2 production). The bioluminescence was not inhibited by cinnamaldehyde and cinnamaldehyde derivatives (data not shown) and these results indicate that the enzymes of *Vibrio harveyi *involved in bioluminescence are not inhibited by cinnamaldehyde or cinnamaldehyde derivatives.

### Effect of cinnamaldehyde and cinnamaldehyde derivatives on AI-2 based QS

**Figure 2:**
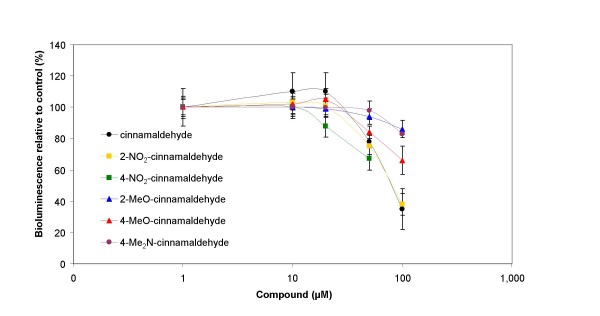
**Effect of cinnamaldehyde and cinnamaldehyde derivatives on AI-2 based QS**. Bioluminescence in *Vibrio harveyi *BB170 as a function of the concentration of cinnamaldehyde and cinnamaldehyde derivatives. Bioluminescence measurements were performed 6 h after the addition of the compounds. Bioluminescence of the control (without addition of compound) was set at 100% and the responses for other samples were normalised accordingly. The error bars represent the standard deviation.

Since bioluminescence is a QS regulated phenotype in *Vibrio harveyi*, we evaluated the effect of the different compounds on bioluminescence in this species. In a first screening we used *Vibrio harveyi *BB170. It was observed that all of the compounds blocked the AI-2 QS system in a concentration-dependent way (Fig. 2). At 100 μM, cinnamaldehyde and 2-NO_2_-cinnamaldehyde were found to be the most active compounds, yielding an inhibition of 65 ± 13% and 62 ± 7%, respectively. 2-MeO-cinnamaldehyde, 4-MeO-cinnamaldehyde and 4-Me_2_N-cinnamaldehyde were found to be less active at this concentration, with inhibitions of 14 ± 5%, 34 ± 9% and 17 ± 1%, respectively. The effect of 4-NO_2_-cinnamaldehyde was only evaluated at lower concentrations because of its growth inhibitory effect. It was found to be the most active compound at concentrations of 25 and 50 μM, with inhibitions of 12 ± 11% and 33 ± 7%, respectively. In general, the QS inhibition assay detected several active QS inhibitors and some striking structure-activity relationships. The inhibitory effect was highly dependent on the substitution pattern of the aromatic ring. Replacement of the dimethylamine (Me_2_N) substituent with a methoxy (MeO) or a nitro (NO_2_) group enhanced the activity. In both the methoxy and the nitro series the activity dropped (approximately ± 10–20%) upon moving the substituent from the para to the ortho position. In general, no cinnamaldehyde derivative was found to be more active than the unsubstituted cinnamaldehyde at concentrations of 100 μM and only one compound, 2-NO_2_-cinnamaldehyde, was found to result in the same level of inhibition. At lower concentrations, 4-NO_2_-cinnamaldehyde was significantly more active than the unsubstituted cinnamaldehyde, but the growth inhibitory effect of this compound prohibited its testing at higher concentrations.

### Effect of cinnamaldehyde and cinnamaldehyde derivatives on the bioluminescence of *Vibrio harveyi *QS mutants

**Figure 3:**
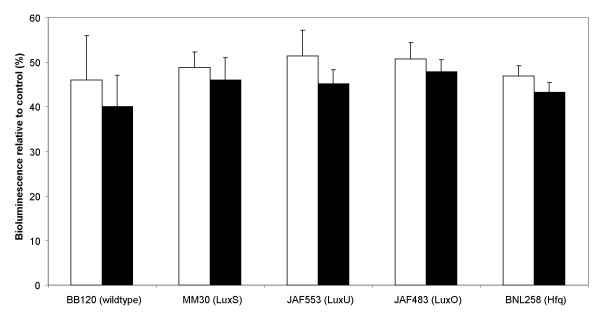
**Effect of cinnamaldehyde and 2-NO_2_-cinnamaldehyde on the bioluminescence of wild type *Vibrio harveyi *BB120 and the different *Vibrio harveyi *QS mutants**. The percentage of bioluminescence of the *Vibrio harveyi *wild type BB120 and the mutants MM30, JAF553, JAF483 and BNL258 with 100 μM cinnamaldehyde (white bars) or 100 μM 2-NO_2_-cinnamaldehyde (black bars) are presented. Measurements were performed 6 h after the addition of the compounds. Bioluminescence of the control (without addition of compound) was set at 100% and the response for the other samples were normalised accordingly. The error bars represent the standard deviation.

Bioluminescence in *Vibrio harveyi *BB170 is mainly controlled by AI-2, as this strain is not responsive to AHL stimulation [7]. Hence we limited the possible target of cinnamaldehyde and cinnamaldehyde derivatives to the AI-2 QS system. To determine the molecular target within the AI-2 QS pathway we measured the effect of cinnamaldehyde and cinnamaldehyde derivatives on the bioluminescence in different QS mutants. *Vibrio harveyi *MM30 has a mutation in the *luxS *gene, making it incapable of producing AI-2. However, this strain will react to exogenously added AI-2 with activation of the QS transduction system leading to bioluminescence. Inhibition of bioluminescence in this mutant would suggest the absence of an inhibitory effect on LuxS. Further we evaluated the effect of the test compounds on the production of AI-2 in *Escherichia coli *K12. The *Vibrio harveyi *JAF553 and JAF483 mutants contain a point mutation in the *luxU *and *luxO *genes, respectively, thereby preventing their phosphorelay capacity. *Vibrio harveyi *BNL258 has a Tn5 insertion in the *hfq *gene, resulting in a non-functional Hfq protein. *Vibrio harveyi *strains JAF553, JAF483 and BNL258 are all constitutively luminescent and inhibition of bioluminescence in one of these indicates that the cinnamaldehyde compounds act downstream of the mutated protein. Cinnamaldehyde and 2-NO_2_-cinnamaldehyde were found to block bioluminescence in *Vibrio harveyi *MM30 (Fig. 3), suggesting that these compounds do not exert their effect at the level of AI-2 production but rather at the level of the QS transduction system. Affirmatively, the supernatants of *Escherichia coli *K12 treated with cinnamaldehyde and cinnamaldehyde derivatives revealed no difference in AI-2 activity compared to the control (data not shown). Cinnamaldehyde and 2-NO_2_-cinnamaldehyde were found to block bioluminescence to the same extent in all other mutants tested (Fig. 3). This suggests that the target of cinnamaldehyde and cinnamaldehyde derivatives is the downstream component of the AI-2 signalling transduction pathway, the transcriptional regulatory protein LuxR.

### Effect of cinnamaldehyde on LuxR protein levels and on LuxR DNA-binding activity

**Figure 4:**
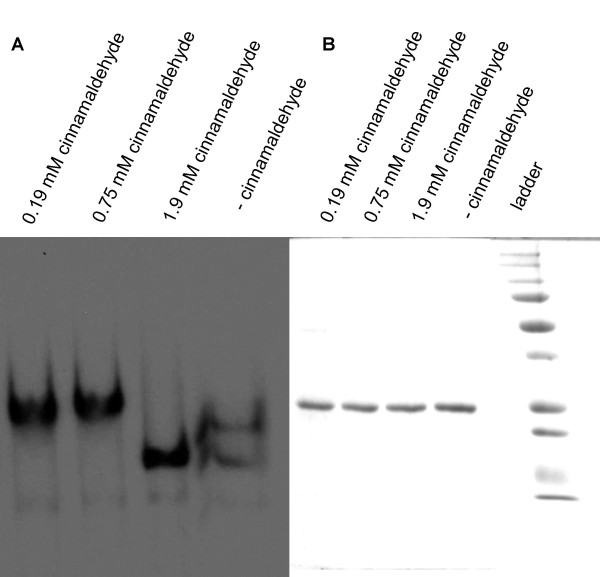
**LuxR DNA-binding as determined by mobility shifts and LuxR protein levels as determined by SDS-PAGE**. A. Autoradiograph after 5% polacrylamide gel electrophoresis of 32P-labelled LuxR promoter DNA containing the LuxR binding sites, mixed with purified LuxR in the presence (0.19, 0.75 and 1.9 mM) and absence of cinnamaldehyde. B. SDS-PAGE of purified LuxR protein in the presence (0.19, 0.75 and 1.9 mM) or absence of cinnamaldehyde.

Using purified LuxR protein, the presence of 0.19 mM and 0.75 mM cinnamaldehyde resulted in a maximal difference in LuxR DNA shift compared to the untreated control (Fig. 4a). These data indicate that *in vitro *binding of the transcriptional regulator LuxR to its promoter sequence is affected in the presence of cinnamaldehyde. Surprisingly, when adding higher concentrations of cinnamaldehyde (1.9 mM) no difference in shift could be observed anymore. This inconsistency was also observed for high furanone concentrations and may be due to aspecific interactions with DNA and/or protein, although the exact reasons for this remain unknown (C. Miyamoto, unpublished data). Purified LuxR was also used to test whether cinnamaldehyde resulted in protein degradation. Three samples of LuxR containing varying amounts of cinnamaldehyde (0.19, 0.75 and 1.9 mM) and an untreated control were stained following electrophoresis on a 10% SDS-PAGE gel and were shown not to have been affected by cinnamaldehyde (Fig. 4b). To test whether the DNA-binding ability was also altered *in vivo*, lysates of *Vibrio harveyi *cells that were grown in the presence and absence of various cinnamaldehyde concentrations were also tested for their ability to cause a mobility shift of LuxR DNA (data not shown). Surprisingly, no effects were observed with concentrations < 1 mM. Using 1 mM cinnamaldehyde, there was about 4-fold less shift of LuxR DNA for the same amount of total protein in the lysate of *Vibrio harveyi *BB120 treated with cinnamaldehyde. There are several possible explanations for this apparent contradiction in terms of cinnamaldehyde concentrations required to cause a band shift. First of all, there may be considerable differences between the extra- and intracellular cinnamaldehyde concentrations, possibly explaining why we observed a shift with 0.19 mM cinnamaldehyde when purified LuxR protein was used but that higher concentrations were required when cell lysates were used. Secondly, there are no data on how much inhibition of binding of LuxR to its promotor is required in order to observe phenotypic changes (e.g. changes in bioluminescence). It may very well be that relatively minor changes in LuxR DNA binding (caused by relatively low cinnamaldehyde concentrations) are sufficient to cause reductions in bioluminescence but would go unnoticed in the gel shift assay. Combined, our data indicate that in the presence of cinnamaldehyde binding of the transcriptional regulator LuxR to its promoter sequence is affected, while leaving the protein intact. However, further research is needed to explain the differences between the *in vitro *and *in vivo *situation in terms of the cinnamaldehyde concentration required to observe this effect. Interestingly, the best-studied QS inhibitors, halogenated furanones, also interfere with binding of LuxR to its promoter sequence without degrading the protein [26].

### Effect of cinnamaldehyde and cinnamaldehyde derivatives on *Vibrio anguillarum *protease and pigment production

**Figure 5:**
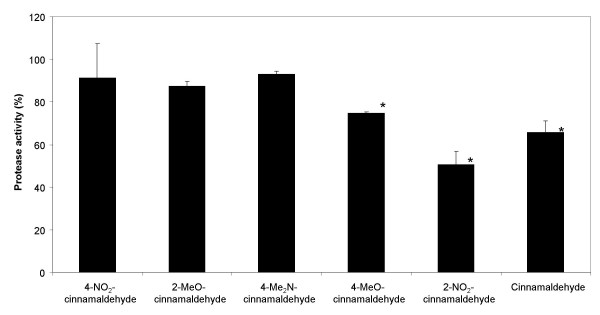
**Effect of cinnamaldehyde and cinnamaldehyde derivatives on the protease activity of *Vibrio anguillarum *LMG 4411**. Cinnamaldehyde and cinnamaldehyde derivatives were tested at 100 μM, except 4-NO_2_-cinnamaldehyde (25 μM). The effect of cinnamaldehyde or cinnamaldehyde derivatives on protease activity was compared to an untreated control. The error bars represent the standard deviation. *: Signal significantly different from the control (p < 0.05).

**Figure 6:**
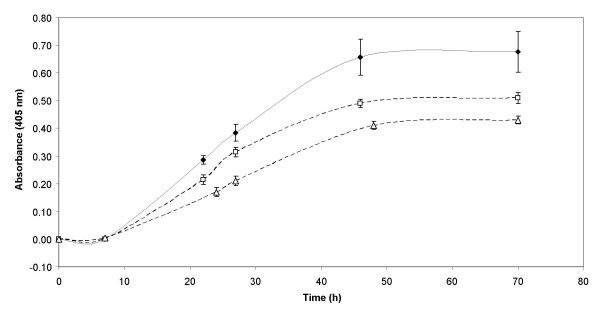
**Effect of cinnamaldehyde and 2-NO_2_-cinnamaldehyde on the pigment production of *Vibrio anguillarum *LMG 4411**. Cinnamaldehyde and 2-NO_2_-cinnamaldehyde were tested at 100 μM. *Vibrio anguillarum *LMG 4411 was allowed to produce pigment in the absence (solid symbol) or presence of cinnamaldehyde (open symbol, square) or 2-NO_2_-cinnamaldehyde (open symbol, triangle). Three ml samples were taken at multiple time points. The effect of cinnamaldehyde or 2-NO_2_-cinnamaldehyde on pigment production was estimated by measuring the absorbance at 405 nm. The error bars represent the standard deviation.

Cinnamaldehyde and 2-NO_2_-cinnamaldehyde were found to decrease protease activity by 34 ± 2% and 49 ± 5%, respectively after 24 h (Fig. 5). 4-MeO-cinnamaldehyde was the only other cinnamaldehyde derivative to cause a significant decrease in protease activity (25 ± 6%) (Fig. 5). A time dependent inhibition of pigment production was found for cinnamaldehyde and 2-NO_2_-cinnamaldehyde. After 48 h, inhibition in pigment production was 25 ± 7% and 40 ± 2% for cinnamaldehyde and 2-NO_2_-cinnamaldehyde (Fig. 6). In contrast, none of the other cinnamaldehyde derivatives were able to significantly reduce pigment production after 48 h (data not shown). Previously, it was shown that several virulence factors in *Vibrio anguillarum*, including pigment and protease production, were regulated by QS. It was found that a mutation in *vanT *(the *luxR *homologue in *Vibrio anguillarum*) resulted in a significant decrease in total protease activity due to loss of expression of the metalloprotease EmpA [16]. Loss of protease activity could have several implications for the virulence of *Vibrio *spp. The protease Vvp of *Vibrio vulnificus*, which is homologous to EmpA, is thought to play an essential role in the colonisation of mucosal surfaces [31]. In addition, EmpA protease from *Vibrio anguillarum *is important for virulence during infection of the Atlantic salmon (*Salmo salar*) and contributes to hemorrhagic skin damage [32,33]. Several other phenotypes, including pigment production, were also found to be affected in a *Vibrio anguillarum vanT *mutant [16].

### Effect of cinnamaldehyde and cinnamaldehyde derivatives on biofilm formation

**Figure 7:**
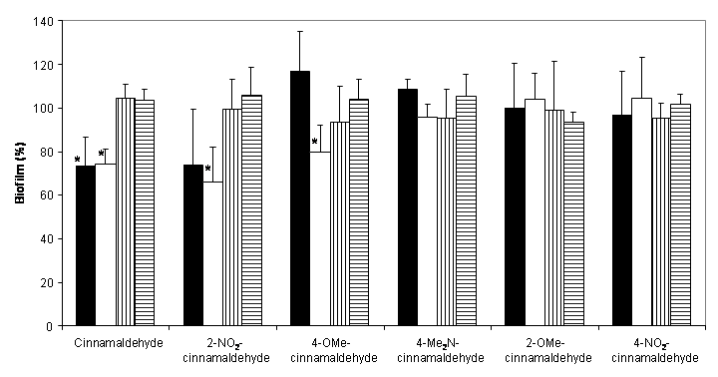
**Effect of cinnamaldehyde and cinnamaldehyde derivatives on *Vibrio *spp. biofilms**. Biomass was quantified through CV staining. Cell-viability was quantified through CTB staining. CV signals are presented as a percentage compared to 100% control not receiving treatment (black bars = *Vibrio vulnificus*; white bars = *Vibrio anguillarum*). CTB signals are presented as a percentage compared to a 100% control not receiving treatment (vertical striped bars = *Vibrio vulnificus*; horizontal striped bars = *Vibrio anguillarum*). *: Signal significantly different compared to 100% control (p < 0.05).

Cinnamaldehyde was previously shown to inhibit *Escherichia coli *biofilms. Since cinnamaldehyde significantly reduced swimming motility in *Escherichia coli *it was hypothesized that reduced biofilm formation could be explained in part by an inability of the strain to reach the substratum [34]. However it should be noticed that no link with QS was described and cinnamaldehyde was used in high concentrations (> 2000 μM). Cinnamaldehyde and some cinnamaldehyde derivatives decreased biofilm formation in *Vibrio anguillarum *LMG 4411 and *Vibrio vulnificus *LMG 16867 (Fig. 7). Cinnamaldehyde reduced total biomass (as measured by crystal violet staining, CV) with 26 ± 7% and 27 ± 13% in *Vibrio anguillarum *LMG 4411 and *Vibrio vulnificus *LMG 16867, respectively. 2-NO_2_-cinnamaldehyde and 4-MeO-cinnamaldehyde resulted in a significant decrease in biomass of *Vibrio anguillarum *LMG 4411 (decrease of 34 ± 16% and 20 ± 12%, respectively). No effect of cinnamaldehyde derivatives on *Vibrio vulnificus *LMG 16867 biomass was observed (Fig. 7). The cell-viability assay revealed no significant decrease in the number of metabolically active cells in *Vibrio anguillarum *LMG 4411 and *Vibrio vulnificus *LMG 16867 biofilm following treatment. In summary, cinnamaldehyde has an effect on total biofilm biomass but not on the number of viable cells. This suggests that cinnamaldehyde may have an effect on the production and/or accumulation of the exopolysaccharide (EPS) matrix (which is also stained with CV). To investigate this hypothesis, EPS was stained using Calcofluor white. Calcofluor white is a fluorescent dye which binds β1–3 and β1–4 carbohydrate linkages and which has been used to study EPS in a variety of organisms [35-37]. The staining was carried out on biofilms treated with cinnamaldehyde as this compound overall had most effect on biofilm biomass as assessed using CV. The use of cinnamaldehyde resulted in a lower fluorescent signal compared to an untreated control (81 ± 13% and 69 ± 27% in *Vibrio anguillarum *LMG 4411 and *Vibrio vulnificus *LMG 16867, respectively). These data support the hypothesis that the effect of cinnamaldehyde on biofilm formation can be explained by reduced EPS production and/or accumulation.

### Protection of Artemia from *Vibrio harveyi*

**Figure 8:**
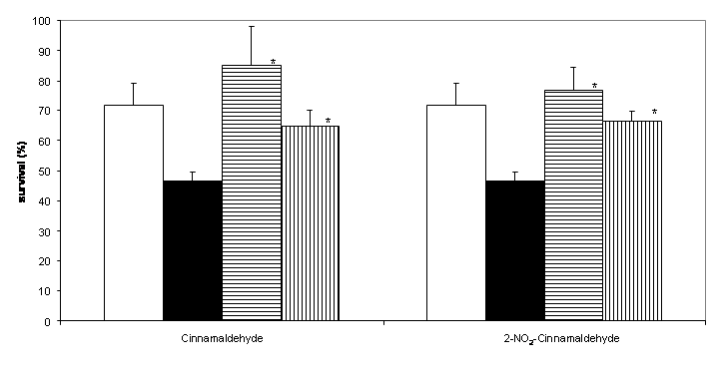
**Effect of cinnamaldehyde and 2-NO_2_-cinnamaldehyde on the survival of *Artemia***. White bars represent the survival of *Artemia *without challenge with *Vibrio harveyi *BB120. Black bars represent the percentage survival of *Artemia *after challenge with *Vibrio harveyi *BB120 in untreated conditions. Striped bars represent the percentage of survival of *Artemia *after challenge with *Vibrio harveyi *BB120 when treated with cinnamaldehyde or 2-NO_2_-cinnamaldehyde (horizontal: 100 μM stripes; vertical stripes: 150 μM, respectively). *: Survival significantly different from the treatment with pathogen alone (p < 0.01).

For many pathogenic *Vibrio *spp., the production of protease, pigment and their capacity to form biofilms contribute to their virulence [31-33]. We investigated the ability of cinnamaldehyde and 2-NO_2_-cinnamaldehyde, the two most active inhibitors, to protect *Artemia *shrimp against the virulent *Vibrio harveyi *BB120 strain. To this end, we followed the survival of *Artemia *after exposure to *Vibrio harveyi *BB120, with and without addition of compounds (Fig. 8). Cinnamaldehyde and 2-NO_2_-cinnamaldehyde alone had no effects on *Artemia *shrimp (data not shown). As expected, high mortality rates were observed when exposing *Artemia *to *Vibrio harveyi *BB120. In contrast, cinnamaldehyde and 2-NO_2_-cinnamaldehyde were able to completely protect *Artemia *against virulent *Vibrio harveyi *BB120 when used at concentrations of 100 μM and 150 μM (Fig. 8). At these concentrations, there was no effect on the growth of *Vibrio harveyi *BB120, ruling out that the protective effect of cinnamaldehyde and 2-NO_2_-cinnamaldehyde was due to inhibition of the bacterial pathogen. These results suggest that cinnamaldehyde and cinnamaldehyde derivatives may be useful as antipathogenic compounds.

### Effect of cinnamaldehyde on the starvation response

**Figure 9:**
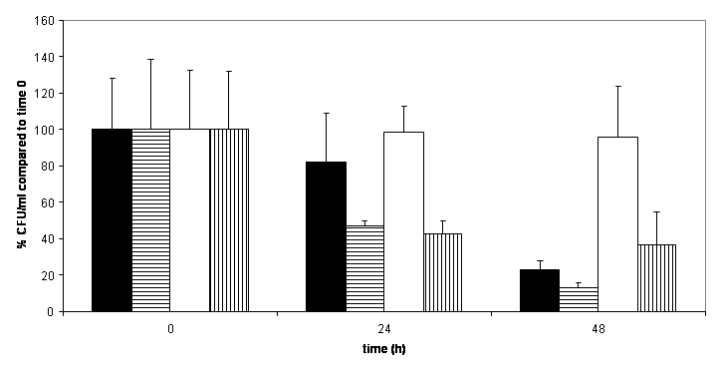
**Effect of cinnamaldehyde on *Vibrio *spp. starvation response**. The cells were allowed to starve in the presence (horizontal striped bars = *Vibrio vulnificus*; vertical striped bars = *Vibrio anguillarum*) and absence (black bars = *Vibrio vulnificus*; white bars = *Vibrio anguillarum*) of cinnamaldehyde. The number of CFU/ml was determined after 24 h and 48 h on TSA plates containing 2% NaCl. Data are presented as a percentage of the initial count. Error bars represent standard deviations.

The effect of cinnamaldehyde on the starvation response of *Vibrio vulnificus *LMG 16867 and *Vibrio anguillarum *LMG 4411 was investigated. In the control experiment no decrease in the number of culturable cells after 24 h of starvation was observed (Fig. 9). Upon treatment with cinnamaldehyde, however, cell numbers were significantly reduced (53 ± 3% and 57 ± 7% for *Vibrio vulnificus *LMG 16867 and *Vibrio anguillarum *LMG 4411, respectively) (p < 0.05). After 48 h, cell numbers were even further reduced in the cinnamaldehyde treated cultures (87 ± 3% and 63 ± 18% for *Vibrio vulnificus *LMG 16867 and *Vibrio anguillarum *LMG 4411, respectively), while there was only a 77 ± 5% and 4 ± 28% reduction in number of culturable cells in the control for *Vibrio vulnificus *LMG 16867 and *Vibrio anguillarum *LMG 4411, respectively. Bacteria are known for their ability to survive and respond to changes in their surroundings. One of these adaptations is the starvation response found in many marine bacteria. *Vibrio *spp. are known to survive for a long time without the addition of supplemental nutrition and this starvation response allows cells to survive adverse conditions. QS is thought to play a role in this response to stress conditions [38]. Our data indicate that inhibition of AI-2 based QS suppresses the starvation response and makes cells more susceptible to starvation-associated stress conditions. This is in agreement with a previously published study [17] in which starvation survival in *Vibrio vulnificus *was reduced by mutation of LuxR in the QS system and by halogenated furanones.

### Effect of cinnamaldehyde on antibiotic susceptibility

**Figure 10:**
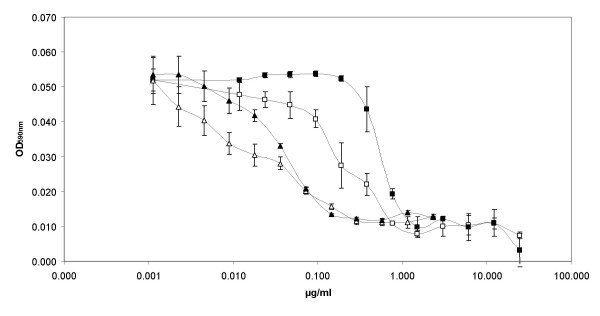
**Effect of cinnamaldehyde on antibiotic susceptibility of *Vibrio vulnificus *LMG 16867**. Effects of chloramphenicol (squares) and doxycycline (triangles) on the growth of *Vibrio vulnificus *LMG16867 in the presence (open symbols) and absence (solid symbols) of cinnamaldehyde (100 μM) are presented. The absorbance at 590 nm was measured after 24 h of growth. Error bars represent standard deviations.

We have examined the association between QS and antibiotic susceptibility in two *Vibrio *spp. Two antibiotics with a different mode of action were chosen. Chloramphenicol, previously used as prophylactic in aquaculture, targets the 50S ribosomal subunit [39,40]. Doxycycline, an antibiotic targeting the 30S ribosomal subunit, is the recommended antibiotic therapy for *Vibrio vulnificus *infections [41]. *Vibrio vulnificus *LMG 16867 showed an increased antibiotic susceptibility when treated with cinnamaldehyde (Fig. 10). This difference was most pronounced when using chloramphenicol. In contrast, in *Vibrio anguillarum *LMG 4411, no differences were observed between cinnamaldehyde treatment and control (data not shown). Previously, it was found that QS inhibition could alter the susceptibility of a strain towards antimicrobial agents. *Vibrio cholerae *strains with various mutations in the AI-2 signal transduction system appeared to be more sensitive to treatment with hydrogen peroxide [42]. Similarly, a *Streptococcus anginosus *LuxS mutant was found to be more susceptible towards ampicillin and erythromycin than the wild type strain [43].

## Conclusion

Cinnamaldehyde and several derivatives were shown to interfere with AI-2 based QS by decreasing the ability of LuxR to bind to its target promoter sequence. These compounds, used in sub-inhibitory concentrations, did not only affect *in vitro *the production of multiple virulence factors and biofilm formation, but also reduced *in vivo *the mortality of *Artemia *shrimp exposed to *Vibrio harveyi *BB120. In addition, cinnamaldehyde reduced the ability to cope with stress factors like starvation and exposure to antibiotics. Our results indicate that cinnamaldehyde and cinnamaldehyde derivatives are potentially useful antipathogenic lead compounds for treatment of vibriosis.

## Methods

### Cinnamaldehyde and cinnamaldehyde derivatives

Cinnamaldehyde (Sigma-Aldrich, Bornem, Belgium) and cinnamaldehyde derivatives [4-MeO-cinnamaldehyde (VWR International, West Chester, USA), 2-MeO-cinnamaldehyde (Wako Pure Chemical Industries, Osaka, Japan), 4-NO_2_-cinnamaldehyde, 2-NO_2_-cinnamaldehyde and 4-Me_2_N-cinnamaldehyde (Acros Organics, Geel, Belgium)] (Fig. 1), were diluted in DMSO (0.5% v/v). The stock solutions were stored at -20°C. Control solutions (CS) contained the same amount of DMSO, without cinnamaldehyde or cinnamaldehyde derivatives.

### Bacterial strains, plasmid and growth conditions

The strains and plasmid used in this study are shown in Table 1. All strains were routinely cultured in Marine-Broth (MB) (BD, Sparks, MD, USA) in the presence of appropriate antibiotics, except for *Escherichia coli *DH5α and K12, which were grown in Luria-Bertani broth (LB) (BD). The medium was supplemented with 100 μg/ml ampicillin (Sigma-Aldrich) for *Escherichia coli *DH5α containing the pBluelux plasmid. *Vibrio anguillarum *LMG 4411, *Vibrio vulnificus *LMG 16867 and the various *Vibrio harveyi *strains were cultured overnight at 30°C on a rotary shaker. *Escherichia coli *DH5α and K12 were cultured overnight at 30°C and 37°C, respectively, without agitation. Minimal inhibitory concentrations were determined for each compound by using a microdilution assay in flat bottomed 96-well microtiter plates (TPP, Trasadingen, Switzerland), using MB and LB medium for all vibrios and both *Escherichia coli *strains, respectively. The plates were incubated for 24 h and the absorption at 590 nm was measured using a Victor Wallac^2 ^multilabel counter (Perkin Elmer Life and Analytical Sciences, Boston, MA, USA).

**Table 1:** Strains and plasmid used in this study.

Strain/plasmid	Relevant features	Reference or source
*Vibrio harveyi *strains		
		
BB120	Wild type from which strains BB152, BB170, MM30, JAF553, JAF483 and BNL258 are derived	[9]
BB170	*luxN*::Tn5	[7]
MM30	*luxS*::Tn5	[10]
JAF553	*luxU *H58A linked to Kan^R^	[48]
JAF483	*luxO *D47A linked to Kan^R^	[14]
BNL258	*hfq*::Tn5*lacZ*	[49]

*Vibrio anguillarum*		
		
LMG 4411	Isolated from young sea trout (*Salmo trutta*)	BCCM/LMG

*Vibrio vulnificus*		
		
LMG 16867	Isolated from tankwater from eelfarm	BCCM/LMG

*Escherichia coli *strains		
		
DH5α	AI-2 ^- ^strain	[23]
K12 ATCC 25404	AI-2^+ ^strain	[23]

Plasmid		
		
pBlueLux	pBluelux polylinker and *luxCDABE*	S. Atkinson

BCCM/LMG: Belgian Co-ordinated Collections of Micro-organisms/Laboratory of Microbiology collection (Ghent University, Belgium); ATCC: American Type Culture Collection

### Effect of cinnamaldehyde and cinnamaldehyde derivatives on bioluminescence

To determine whether any of the compounds had an effect on bioluminescence not related to inhibition of QS, *Escherichia coli *DH5α was transformed with the pBluelux plasmid, containing *luxCDABE *under control of a *lacZ *promoter and the effect on bioluminescence was measured. The pBluelux plasmid was transformed in *Escherichia coli *DH5α as follows. Overnight cultures were suspended in a 50 mM CaCl_2 _solution at 0°C. The pBluelux plasmid was added and the solution was incubated for 15 min. After this, the solution was transferred to 42°C for 90 sec and the cell suspension was plated on Trypton soy agar (TSA) (Oxoid, Basingstoke, Hampshire, UK) containing 100 μg/ml ampicillin (Sigma-Aldrich) for selection of transformants. For the bioluminescence assay an overnight culture was diluted to OD_590 nm _of approximately 0.1 and 100 μl of cell suspension was added to each well of a black 96-well microtiter plate (Perkin Elmer). The effect on bioluminescence for the active compounds was compared to controls not receiving the active molecules.

### Bioassay for LuxS inhibition

In order to determine whether any of the compounds tested had an effect on production of AI-2, AI-2 activity was measured in supernatants of *Escherichia coli *K12 cultures grown for 16 h with or without compounds. Overnight cultures of *Escherichia coli *K12 were centrifuged (5000 rpm, 5 min, room temperature) and filter sterilised (0.22 μm, Whatman GmbH, Dassel, Germany). The supernatants were used immediately or stored at -20°C. AI-2 levels were determined in a *Vibrio harveyi *BB170 assay as described previously [21]. In brief, an overnight culture of the reporter strain was diluted 1:5000 into fresh sterile MB medium and 90 μl of this cell suspension was added to the wells of a black 96-well microtiter plate (Perkin Elmer). Ten μl of the appropriate sterile supernatants was then added to the wells, the microtiter plates were incubated at 30°C and bioluminescence was measured hourly using a Wallac Victor^2 ^multilabel counter (Perkin Elmer). Bioluminescence was expressed as the fraction of bioluminescence measured in the positive control reaction. Confirmation of these results was obtained using *Vibrio harveyi *MM30, a Δ LuxS mutant, instead of *Vibrio harveyi *BB170. The effect on bioluminescence for the active compounds was compared to controls not receiving the active molecules.

### Other *Vibrio harveyi *bioassays

Using *Vibrio harveyi *strains BB120, JAF553, JAF483 and BNL258, we determined whether the molecular target of our compounds was located in the AI-2 signalling transduction pathway. The bioluminescence assay as described above was used with minor modifications. In brief, the positive control reaction received 10 μl of *Escherichia coli *K12 supernatant, without addition of the test molecule. Negative control reactions received 10 μl sterile MB-medium. Other wells received 10 μl of *Escherichia coli *K12 supernatants (containing AI-2) and appropriate amounts of the test molecule.

### Effect of cinnamaldehyde on LuxR protein levels and DNA-binding activity

Mobility shift assays and SDS-PAGE assays were performed as described previously [26] with minor modifications. *Vibrio harveyi *BB120 cells were grown in the presence and absence of cinnamaldehyde and all cell lysates were taken at different optical densities (OD_600 nm _= 1.2, 1.6, 1.8 and 2.1). Previously purified LuxR [44] was used for mobility shift and SDS-PAGE assay. For SDS-PAGE the following protein standard (Bio-rad) was used: 250, 150, 100, 75, 50, 37, 25, 20, 15 and 10 kDa.

### Quantification of protease activity

*Vibrio anguillarum *LMG 4411 was grown overnight in MB. Protease activity was quantified following inoculation of cultures into medium containing 2.0% Bacto agar (Oxoid), 2.0% NaCl (Novolab, Geraardsbergen, Belgium) and 3.0% Skim Milk powder (Oxoid). Appropriate amounts of test compounds and CS were added to the mixtures, 0.5 ml of these mixtures was added to the wells of a 24-well microtiter plate (TPP, Trasadingen, Switzerland) and the plate was incubated at 30°C. Clearing was measured spectrophotometrically with a Wallac Victor^2 ^multilabel counter after 24 h.

### Quantification of pigment production

*Vibrio anguillarum *LMG 4411 was grown overnight at 30°C in MB. The overnight culture was then diluted to OD_590 nm _= 0.05 in Tryptone Soy Broth (TSB) (Oxoid) containing 5 mM L-tyrosine (Sigma-Aldrich) with or without test compound and incubated at 30°C with shaking. At various time points, 3 ml samples were taken from the cultures and supernatants were collected by centrifugation (5000 rpm, 4 min, room temperature), followed by filter sterilisation (0.22 μm). Pigment production was followed by measuring the absorbance at 405 nm.

### Biofilm formation assay

*Vibrio anguillarum *LMG 4411 (doubling time T_d_: 3.2 h) and *Vibrio vulnificus *LMG 16867 (T_d_: 5.3 h) were grown overnight in MB, centrifuged, resuspendend in double concentrated Marine Broth (2xMB) and diluted to an OD_590 nm _= 0.1 in 2xMB. Fifty μl of the diluted bacterial suspension was transferred to the wells of a round-bottomed 96-well microtiter plate (TPP). Negative controls received 50 μl of CS. Positive controls received 50 μl of the test compound in appropriate concentrations. Bacteria were allowed to adhere and grow without agitation for 4 h at 30°C. After 4 h, plates were emptied and washed with sterile physiological saline (PS). After this washing step, negative control wells were filled with 50 μL 2xMB and 50 μl CS. Other wells were filled with 50 μl 2xMB and 50 μl compound solution and the plate was incubated for 24 h at 30°C. Biofilm biomass was quantified by crystal violet (CV) staining, as described previously [45]. In brief, plates were rinsed with sterile PS, biofilms were fixed by adding 100 μl 99% methanol for 15 min, after which the methanol was removed and plates were air-dried. Biofilms were then stained with 100 μl CV (Pro-lab Diagnostics, Richmond Hill, ON, Canada). After 20 min, CV was removed and wells were filled with 150 μl 33% acetic acid (Sigma-Aldrich). The absorbance was measured at 590 nm using a Wallac Victor^2 ^multilabel counter and results were expressed as the percentages compared to the signal of the control not receiving treatment. For quantification of the number of metabolically active (i.e. living) cells in the biofilm, a resazurin assay was used [45]. In brief, wells were rinsed after 24 h biofilm formation and 100 μl PS was added, followed by addition of 20 μl CellTiter-Blue (CTB) (Promega, Leiden, The Netherlands) solution. After 60 min, fluorescence (ex_560 nm_/em_590 nm_) was measured using a Wallac Victor^2 ^multilabel counter. For the quantification of EPS, a Calcofluor white staining (Sigma-Aldrich) was used. In brief, wells were rinsed after 24 h biofilm formation and 100 μl phosphate buffered saline (PBS) containing 0.5 μl 5 mM CFW was added to the wells. After 60 min, fluorescence (ex_405 nm_/em_500 nm_) was measured using a Wallac Victor^2 ^multilabel counter.

### *Artemia *Challenge tests

All experiments were performed with high quality hatching cysts of *Artemia franciscana *(EG^® ^Type, batch 6940, INVE Aquaculture, Baasrode, Belgium). 200 mg of cysts were hydrated in 18 ml of tap water during 1 h. Sterile cysts and nauplii were obtained via decapsulation as described previously [46]. Challenge tests were performed as described previously [18] with minor modifications. Briefly, after hatching, groups of 20 nauplii were transferred to new sterile 50 ml tubes that contained 20 ml of 0.22 μm filtered and autoclaved artificial seawater. *Vibrio harveyi *BB120 was washed in filtered and autoclaved artificial seawater after incubation and added to the *Artemia *culture water at a concentration of approximately 10^5 ^CFU/ml. A suspension of autoclaved LVS3 bacteria in filtered and autoclaved artificial seawater was added as feed in a concentration of approximately 10^7 ^CFU/ml culture water. After the addition of 100 μM or 150 μM of cinnamaldehyde or 2-NO_2_-cinnamaldehyde (or an appropriate volume of solvent), the falcon tubes were put back on the rotor and kept at 28°C. *Artemia *cultures to which only autoclaved LVS3 bacteria were added were used as controls. The survival of *Artemia *was scored 48 h after the addition of the strains. All manipulations were done under a laminar flow hood in order to maintain sterility of the cysts and nauplii. Each treatment was done in triplicate.

### Starvation assay

*Vibrio anguillarum *LMG 4411 and *Vibrio vulnificus *LMG 16867 strains were grown overnight in MB, the cells were collected by centrifugation (5000 rpm, 4 min), washed in PS and resuspended in artificial seawater (ASW) [47] containing 0.1% MB (with and without test compound). These suspensions were incubated at 30°C without shaking. At various time points, 1 ml samples were taken and the number of culturable cells was determined by plating serial dilutions on TSA (Oxoid) plates containing 2% NaCl. Results were expressed as the percentage survival compared to the untreated control.

### Effect of cinnamaldehyde on antibiotic resistance

Fifty μL of double concentrated TSB (2xTSB) containing 4% NaCl with or without chlorampenicol (Sigma-Aldrich) or doxycycline (Sigma-Aldrich) (added in the range of 0.001 μg/ml – 25 μg/ml) were dispensed into flat-bottomed 96-well microtiter plates (TPP). An equal amount of cinnamaldehyde was added (final concentration of 100 μM). For the controls, equal amounts of CS were added to the wells. *Vibrio vulnificus *LMG 16867 or *Vibrio anguillarum *LMG 4411 was added in a final concentration of 10^5 ^CFU/ml. The plates were incubated overnight at 30°C and growth was evaluated after 24 h by absorbance measurements at 590 nm using a Wallac Victor^2^multilabel counter.

### Statistics

Independent samples t-tests were performed using the SPSS software, version 15.0 (SPSS, Chicago, IL, USA).

## Authors' contributions

GB carried out most of the experiments, analysed the data, provided figures and tables and drafted the manuscript. TD and PB carried out the in vivo virulence assay. CM carried out the mobility shift and SDS-PAGE assays. SVC coordinated and participated in the selection of the compounds. TC and HN coordinated the study and helped to draft the manuscript. All authors read and approved the final manuscript.
